# Supplementary material for: Structural basis of ligand recognition and design of antihistamines targeting histamine H4 receptor
Source: Nat Commun. 2024 Mar 20;15:2493. doi: 10.1038/s41467-024-46840-5 (PMC10954740; doi:10.1038/s41467-024-46840-5)
Supplement: Supplementary file 1 — Supplementary Information [file 41467_2024_46840_MOESM1_ESM.pdf]

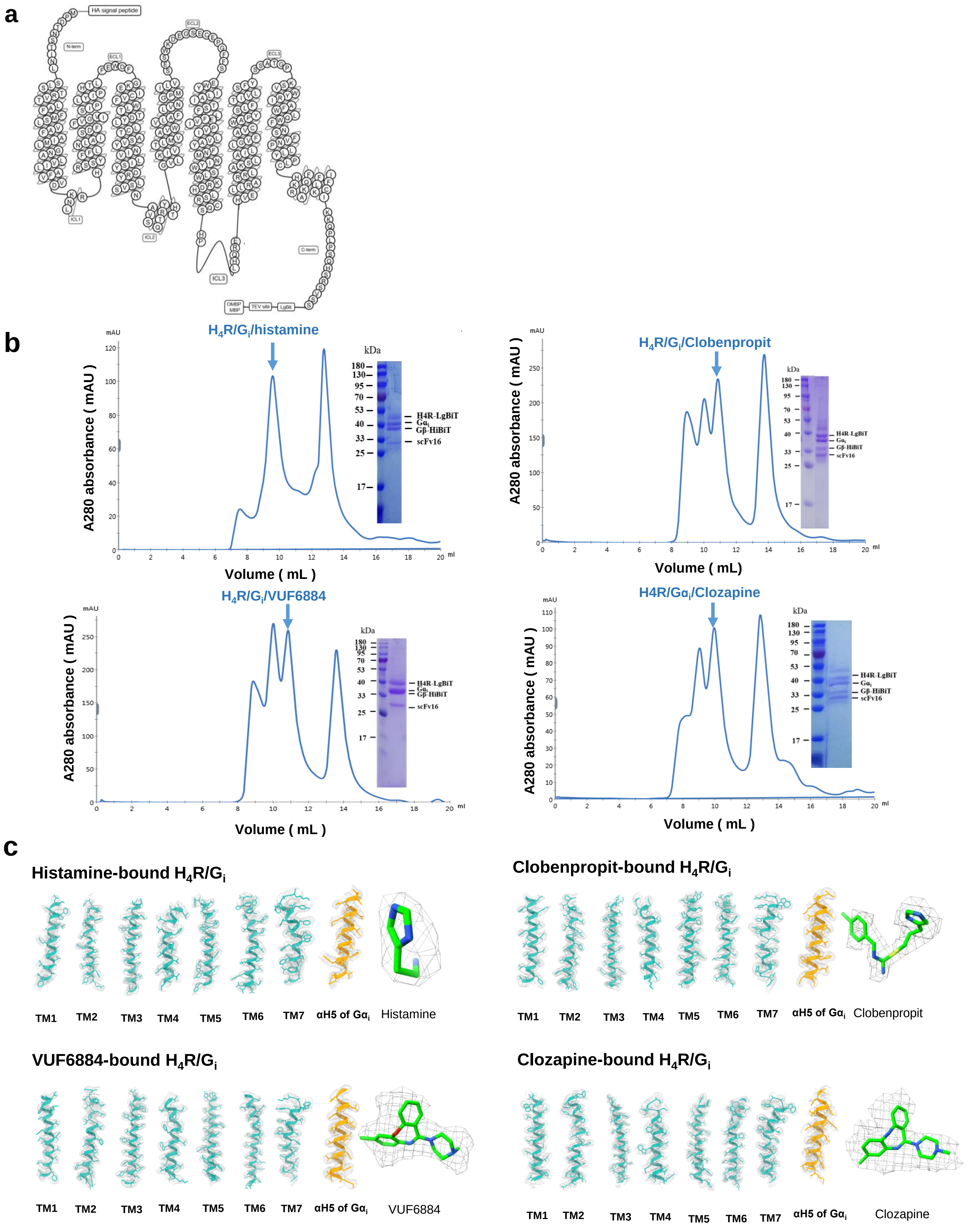

Supplementary Fig. 1 Expression and purification of H_4R. a, A snake-shape diagram of the H_4R construct used in complex assembling, the diagram was adopted from GPCRdb. **b,** Size exclusion column profiles and SDS-PAGE analysis of H_4R/G_i complexes. Source data are provided as a Source Data file. **c,** Cryo-EM density map of representative regions of H_4R/G_i complexes. Map level was set to 0.08 in chimeraX.

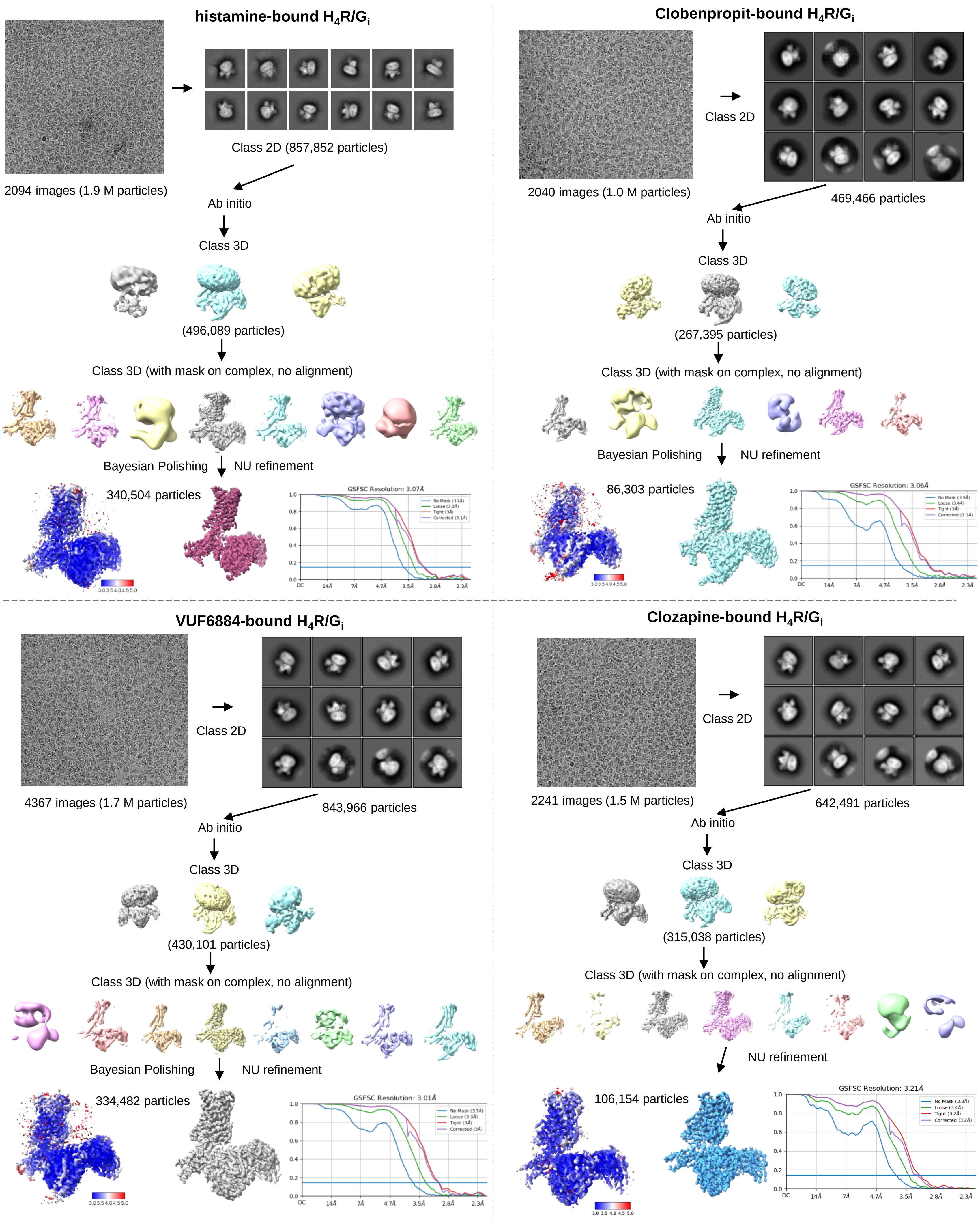

Supplementary Fig. 2 Flow-chart of cryo-EM data process of H_4R/G_i complexes. The image process, 2D, 3D classification and Bayesian Polishing were done by Relion, others were done by CryoSparc.

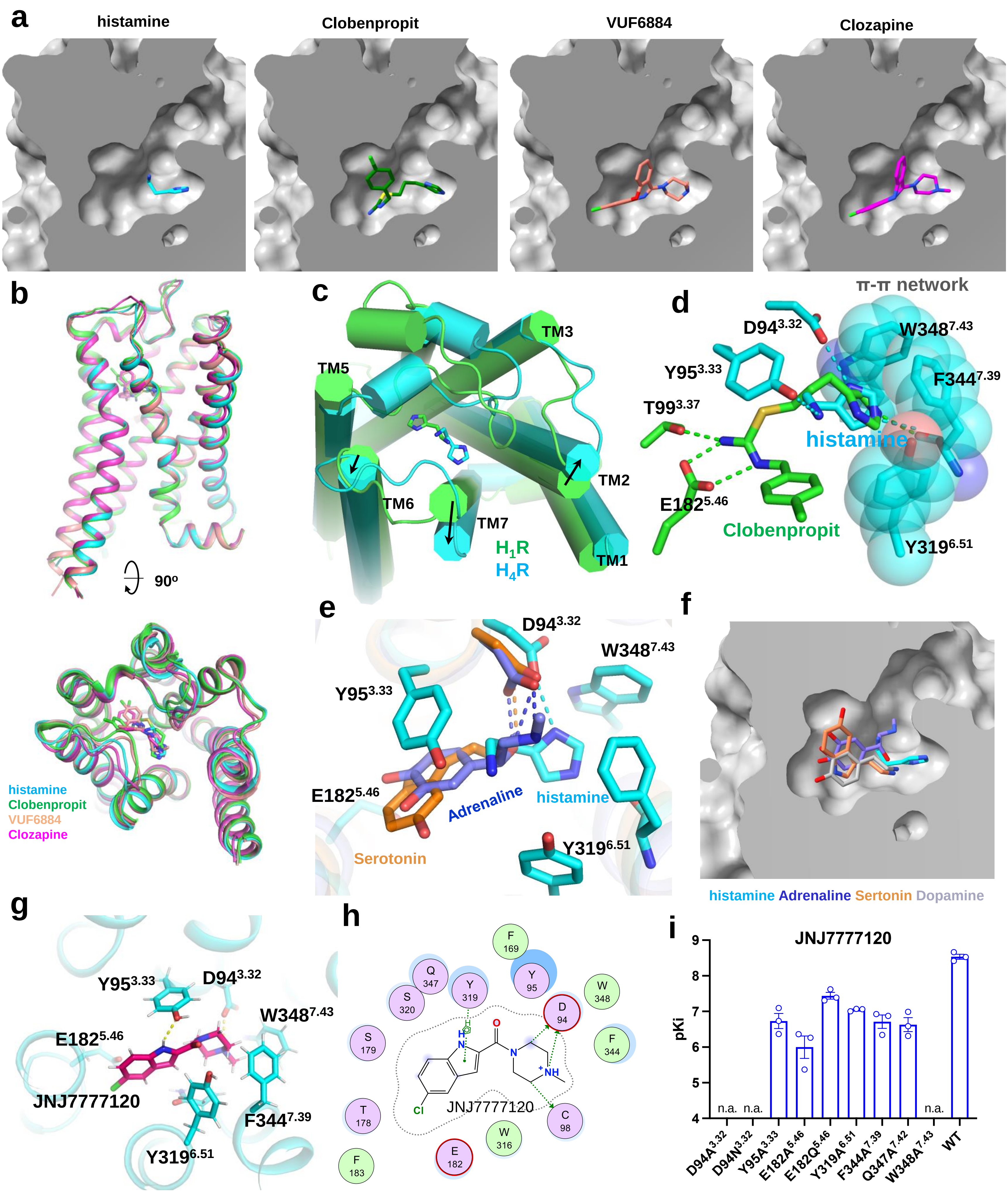

Supplementary Fig. 3 Additional information of the ligand binding pocket of H_4R. **a,** The clipping view of the ligand binding pocket bound with histamine, Clobenpropit, VUF6884 and Clozapine. **b,** An comparison of the overall structures of histamine, Clobenpropit, VUF6884 and Clozapine-bound H_4R. **c,** A comparison of histamine-bound H_1R and H_4R from the top view. **d,** A comparison of ligand/receptor interaction of the histamine-bound H_4R with the Clobenpropit-bound H_4R. **e,** A comparison of adrenaline (PDB: 4ldo) binding in β_2AR and serotonin binding in 5-HT_1A (PDB: 7e2y) with that of histamine in H_4R. **f,** The clipping view of the ligand positions of histamine, dopamine, serotonin, adrenaline in the context of H_4R. **g,** Docking of JNJ7777120 into H_4R. **h,** The ligand/receptor interaction of the docked JNJ7777120 in H_4R. **i,** pKi of H_4R mutants in binding of JNJ777120. Data are presented as mean values ± SEM.; n =3 independent samples. Source data are provided as a Source Data file.

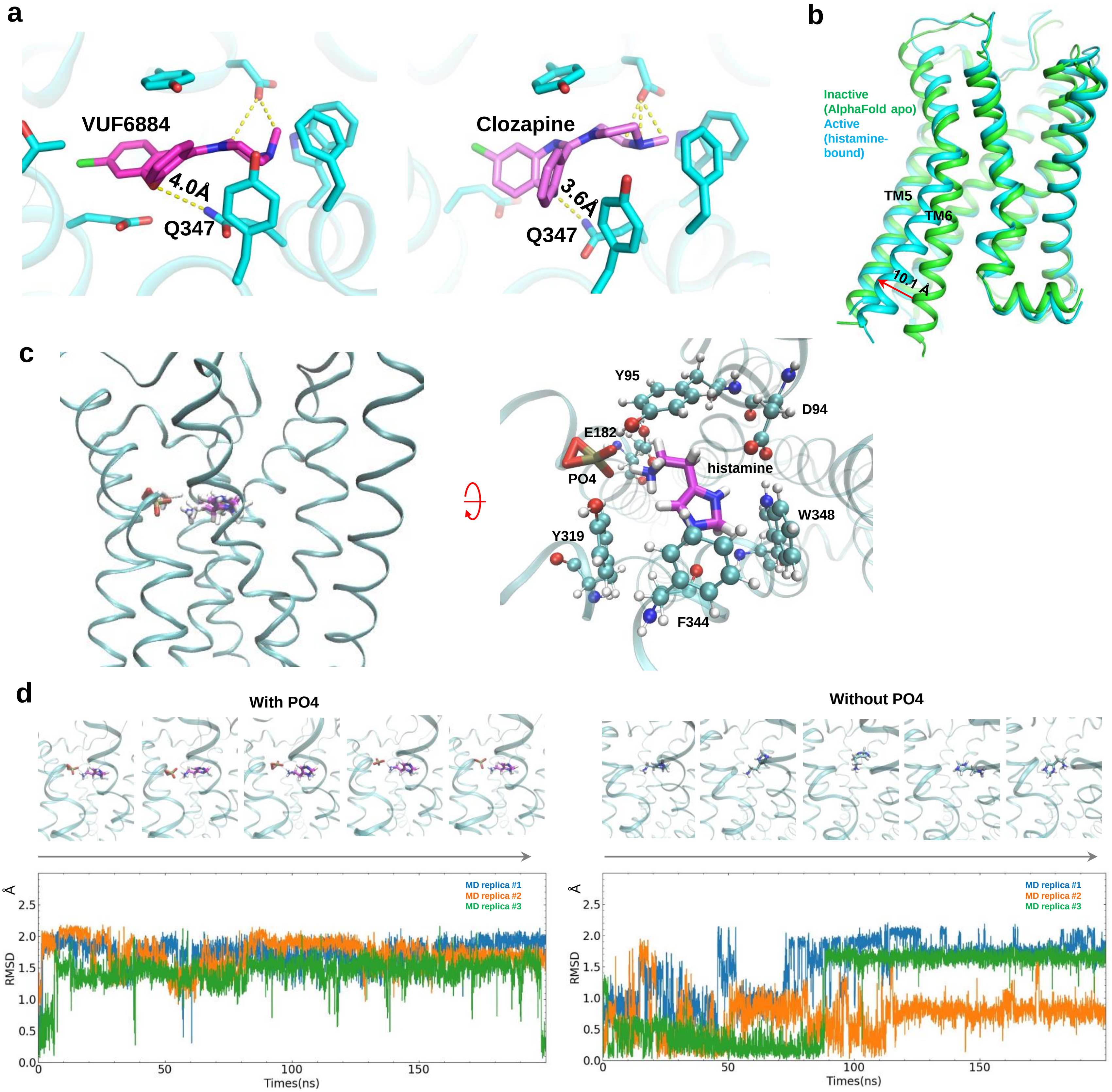

Supplementary Fig. 4 Additional information of H_4R ligand bidning. a, A comparison of VUF6884 and clozapine binding of H4R, focusing on Q347. **b,** A comparison of histamine-bound H4R with the AlphaFold prediction of apo H4R. **c,** A snapshot of MD simulations of histamine PO4 co-binding of H4R. Left panel, the translucent white color indicates the original positions of histamine and PO4 in the cryo-EM structure. **d,** The trajectory analysis of MD simulations of histamine binding with or without the anion PO4 for H4R. The upper panels are snapshots of histamine binding with PO4 (left) and without PO4 (right) in sequential order during the 200 ns simulations. The lower panels are RMSD analysis of histamine during the 200 ns simulations.

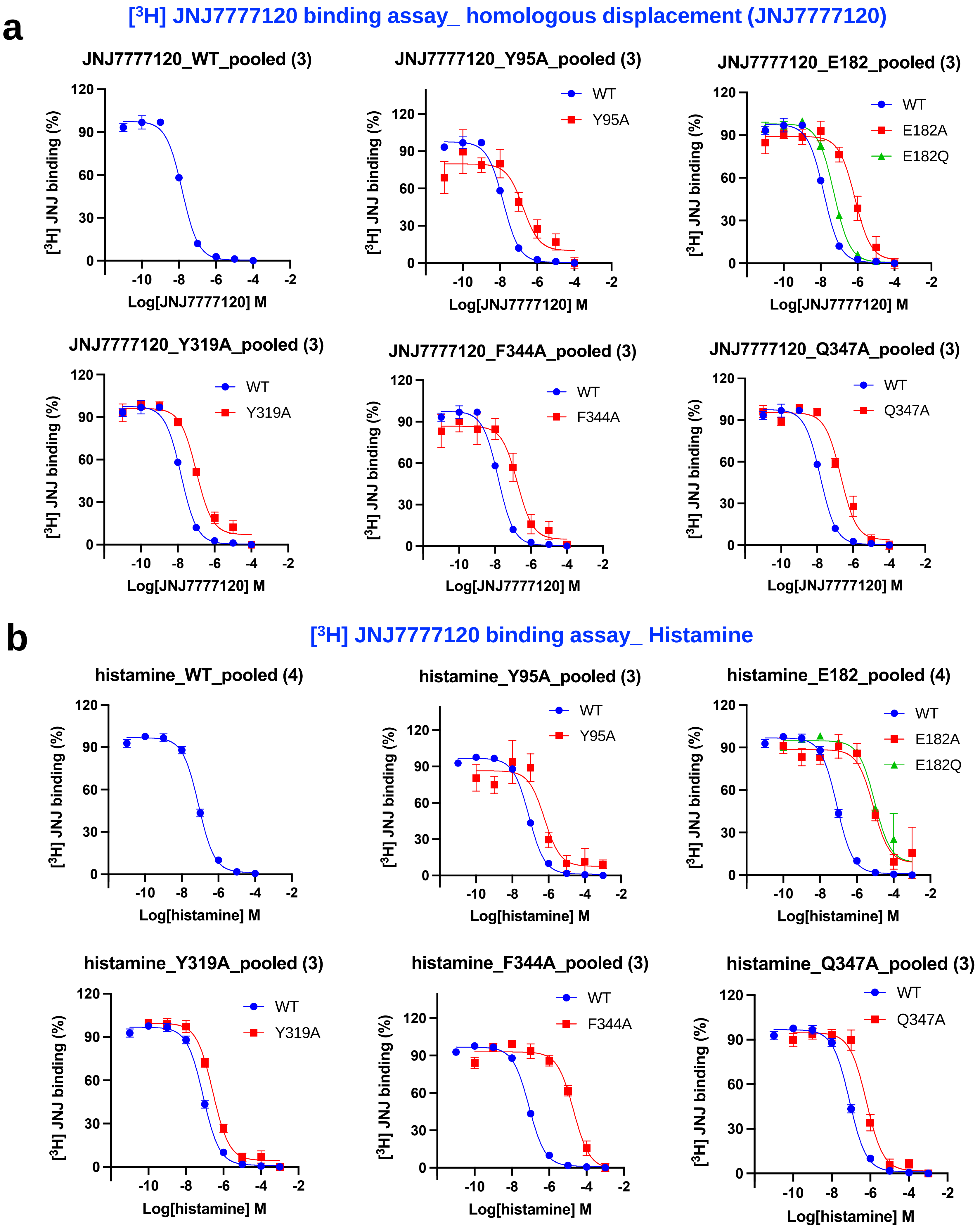

Supplementary Fig. 5 Ligand binding assays of H_4R mutants. a, [3H] JNJ7777120 binding assay via homologous displacement. **b,** The competition binding assay of histamine to [3H] JNJ7777120. Data are presented as mean values ± SEM.; n =3-4 independent samples. Source data are provided as a Source Data file.

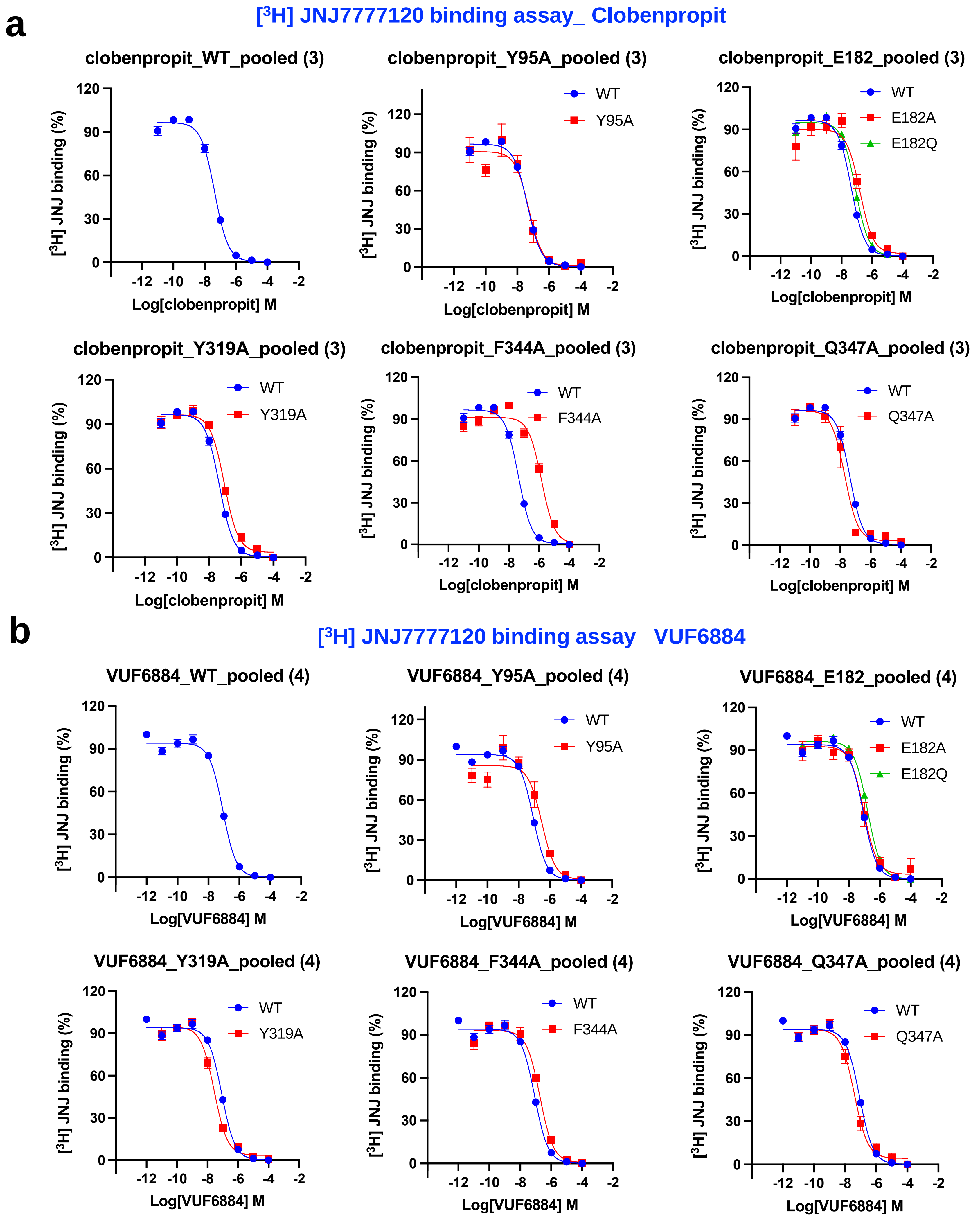

Supplementary Fig. 6 Ligand binding assays of H_4R mutants. a-b, The competition binding assay of Clobenpropit and VUF9884, respectively, to [^{3}H] JNJ7777120. Data are presented as mean values ± SEM.; n =3-4 independent experiments. Source data are provided as a Source Data file.

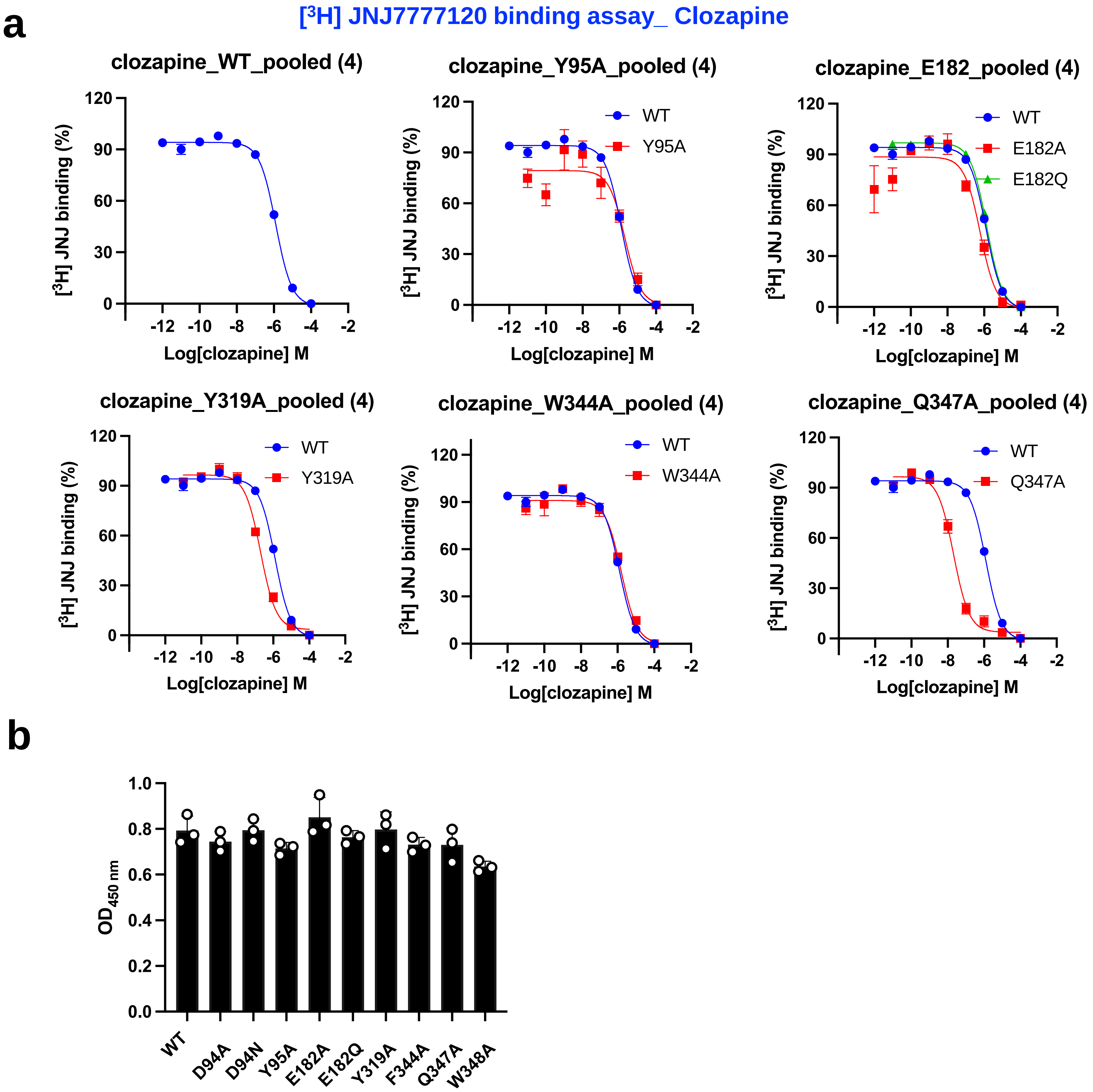

Supplementary Fig. 7 Ligand binding assays of H_4R mutants. a, The competition binding assay of Clozapine to [^{3}H] JNJ7777120. **b,** The surface expression of H_4R mutants (single experiment). Data are presented as mean values ± SEM.; n =4 independent experiments. Source data are provided as a Source Data file.

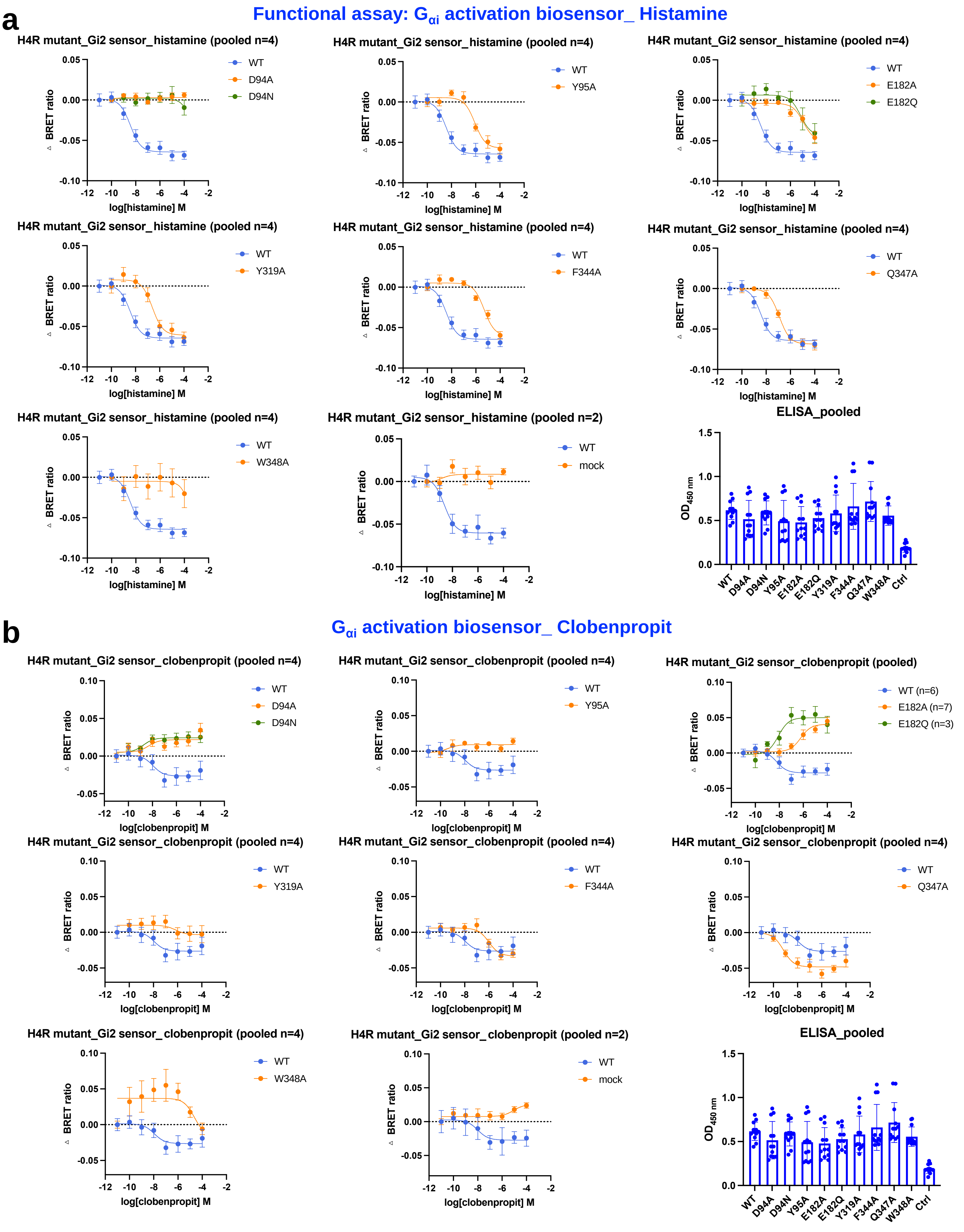

Supplementary Fig. 8 The BRET G_i activation assays of H_4R mutants. a-b, The $G\alpha_i$ biosensor BRET assays of H_4R activation via histamine and Clobenpropit, respectively. Data are presented as mean values ± SEM.; n =3-7 independent experiments, n=2 for mock control experiment. Source data are provided as a Source Data file.

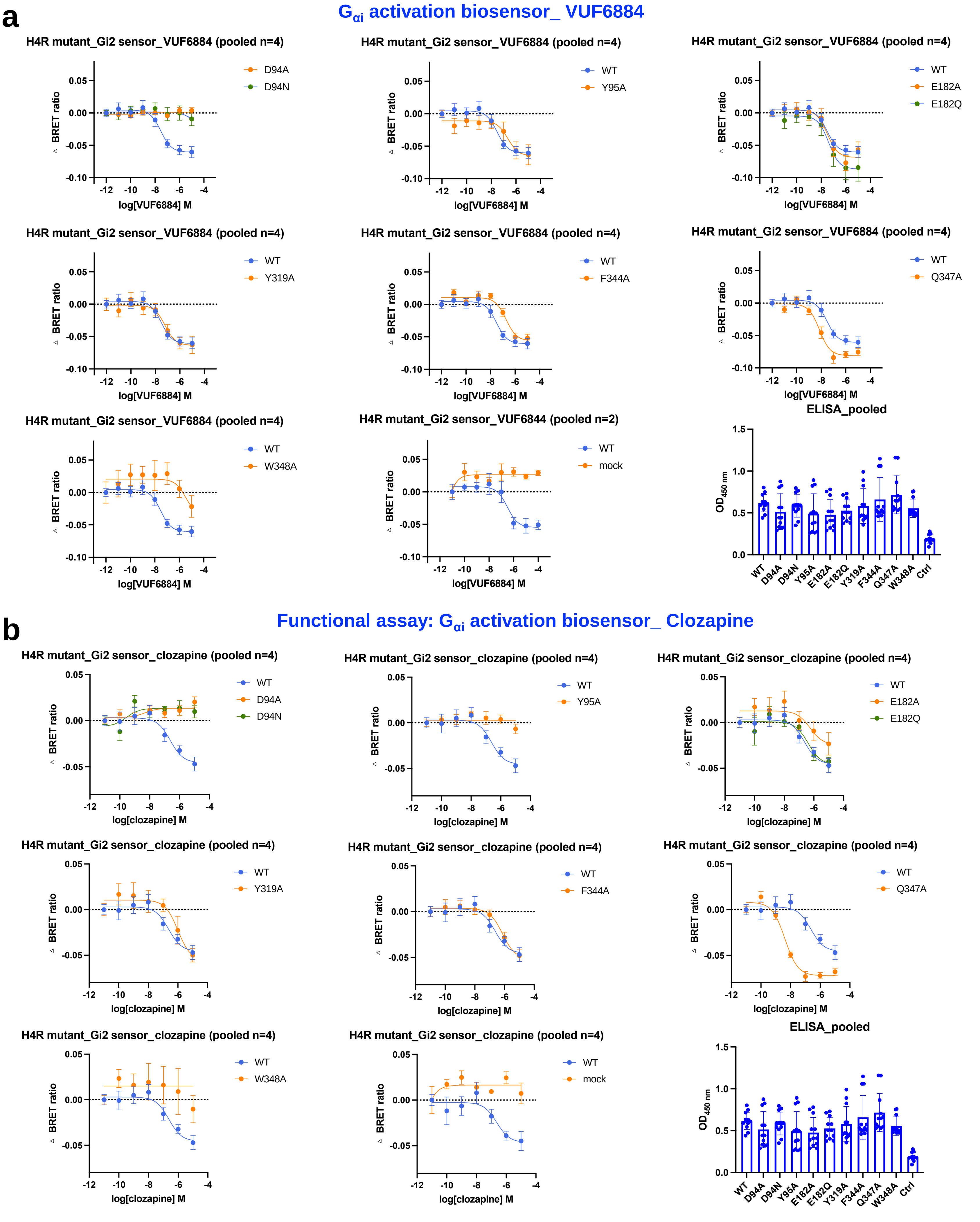

Supplementary Fig. 9 The BRET G_i activation assays of H_4R mutants. a-b， The $G\alpha_i$ biosensor BRET assays of H_4R activation via VUF9884 and Clozapine, respectively. Data are presented as mean values ± SEM.; n =3-4 independent experiments, n=2 for mock control experiment. Source data are provided as a Source Data file.

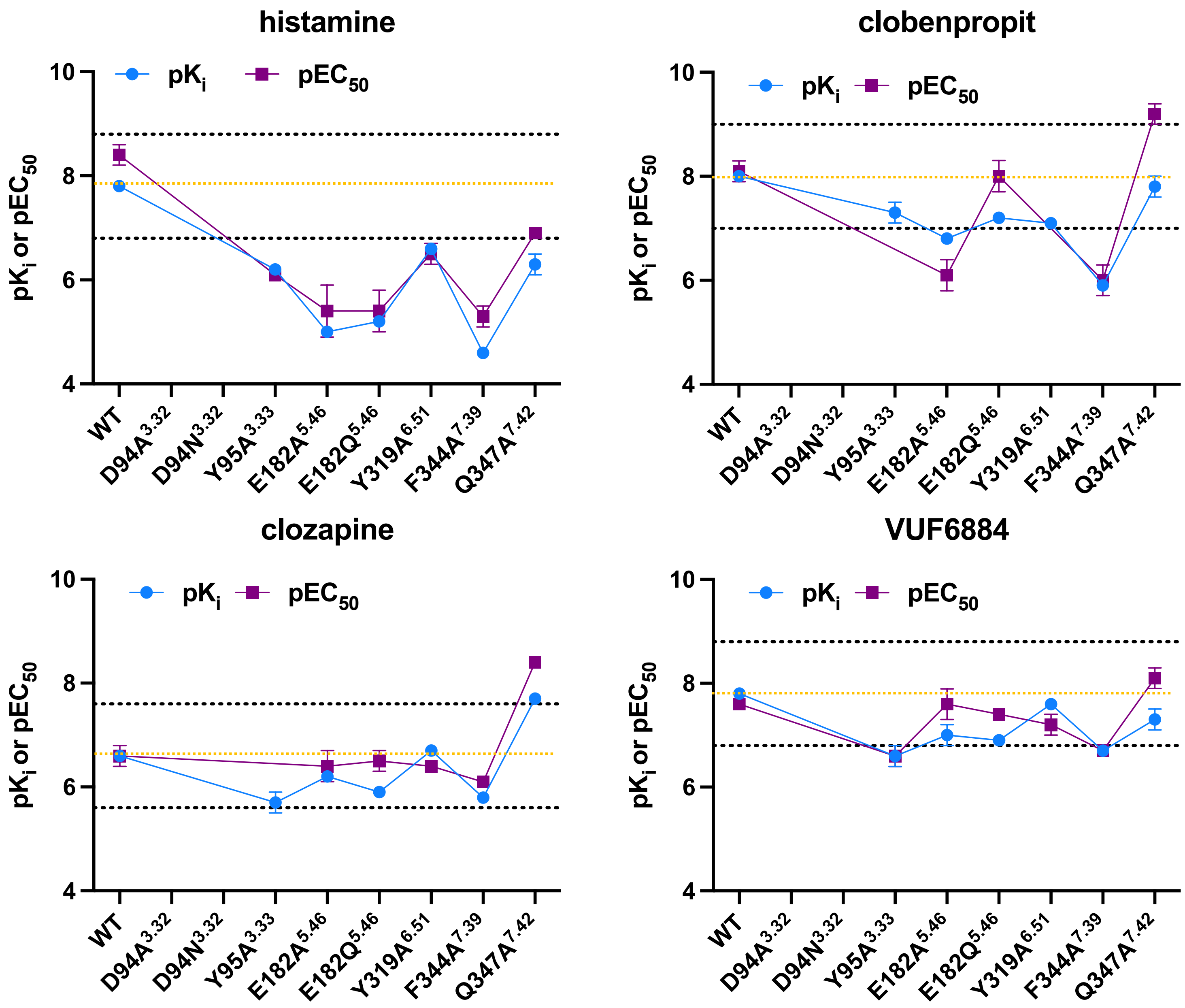

Supplementary Fig. 10 An overview comparison between the RA binding and functional assay. Data are presented as mean values ± SEM.; n =3-7 independent experiments. Source data are provided as a Source Data file.

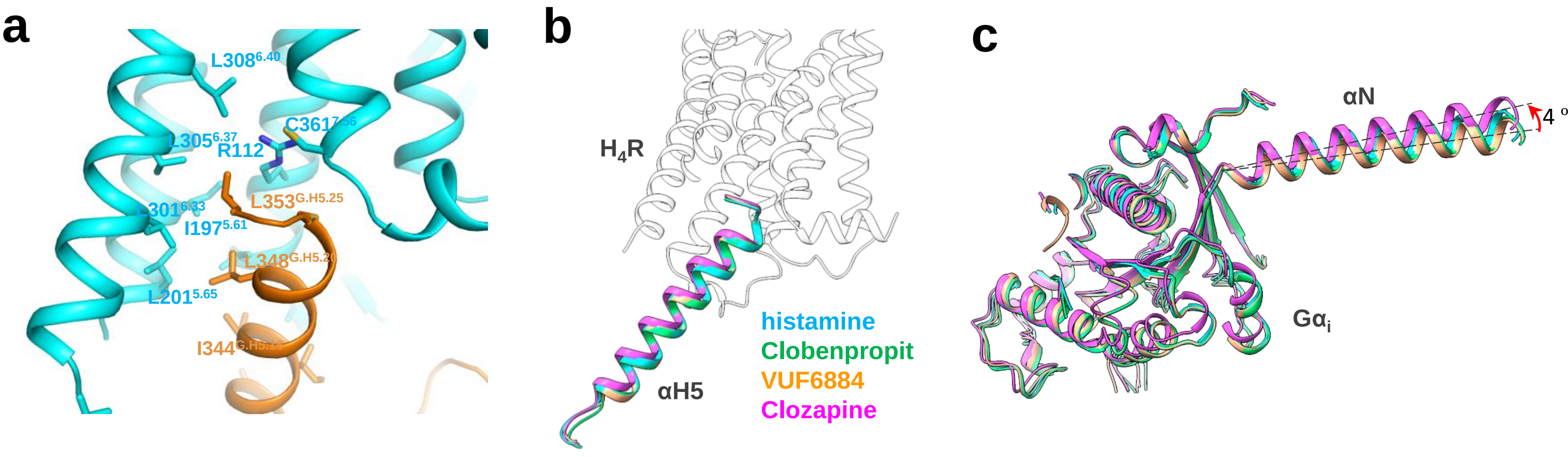

Supplementary Fig. 11 Additional information of H_4R/G_i engagement. a, The details of the hydrophobic interaction between aH5 and TM5-TM6 of the receptor. **b,** A comparison of the αH5/receptor interaction of the histamine, Clobenpropit, VUF9884 and Clozapine-bound H_4R/G_i complex. **c,** A comparison of the αN angles of of the histamine, Clobenpropit, VUF9884 and Clozapine-bound H_4R/G_i complex.

Supplementary Table 1. MD simulation system setting

Box dimensions (Å)	Total atoms	Total waters	Salt concentration	Lipid composition
65 x 65 x 111	44795	27989	KCl, 150 mM	POPC:cholesterol = 4:1
