# Supplementary material for: Structural basis of ligand recognition and design of antihistamines targeting histamine H4 receptor
Source: Nat Commun. 2024 Mar 20;15:2493. doi: 10.1038/s41467-024-46840-5 (PMC10954740; doi:10.1038/s41467-024-46840-5)
Supplement: Supplementary file 3 — Description of Additional Supplementary Files [file 41467_2024_46840_MOESM3_ESM.pdf]

File name: Supplementary Movie 1

Description: Movie track of MD simulation of histamine-bound H_4R. The length is 200ns, interval is 5 steps.
