# Supplementary material for: Structural basis of ligand recognition and design of antihistamines targeting histamine H4 receptor
Source: Nat Commun. 2024 Mar 20;15:2493. doi: 10.1038/s41467-024-46840-5 (PMC10954740; doi:10.1038/s41467-024-46840-5)
Supplement: Supplementary file 5 — Reporting Summary [file 41467_2024_46840_MOESM5_ESM.pdf]

nature portfolio

Corresponding author(s): Yuanzheng He

Last updated by author(s): Mar 11, 2024

# Reporting Summary

Nature Portfolio wishes to improve the reproducibility of the work that we publish. This form provides structure for consistency and transparency in reporting. For further information on Nature Portfolio policies, see our Editorial Policies and the Editorial Policy Checklist.

## Statistics

For all statistical analyses, confirm that the following items are present in the figure legend, table legend, main text, or Methods section.

| n/a | Confirmed | |
|---|---|---|
| ☐ | ☒ | The exact sample size (*n*) for each experimental group/condition, given as a discrete number and unit of measurement |
| ☐ | ☒ | A statement on whether measurements were taken from distinct samples or whether the same sample was measured repeatedly |
| ☐ | ☒ | The statistical test(s) used AND whether they are one- or two-sided<br>*Only common tests should be described solely by name; describe more complex techniques in the Methods section.* |
| ☒ | ☐ | A description of all covariates tested |
| ☒ | ☐ | A description of any assumptions or corrections, such as tests of normality and adjustment for multiple comparisons |
| ☐ | ☒ | A full description of the statistical parameters including central tendency (e.g. means) or other basic estimates (e.g. regression coefficient) AND variation (e.g. standard deviation) or associated estimates of uncertainty (e.g. confidence intervals) |
| ☐ | ☒ | For null hypothesis testing, the test statistic (e.g. *F*, *t*, *r*) with confidence intervals, effect sizes, degrees of freedom and *P* value noted<br>*Give P values as exact values whenever suitable.* |
| ☒ | ☐ | For Bayesian analysis, information on the choice of priors and Markov chain Monte Carlo settings |
| ☒ | ☐ | For hierarchical and complex designs, identification of the appropriate level for tests and full reporting of outcomes |
| ☒ | ☐ | Estimates of effect sizes (e.g. Cohen's *d*, Pearson's *r*), indicating how they were calculated |

*Our web collection on statistics for biologists contains articles on many of the points above.*

## Software and code

Policy information about availability of computer code

| | |
|---|---|
| Data collection | EPU-v2.2.0 |
| Data analysis | Relion 3.1, CryoSPARC 4.3.1, Pymol 2.3.2, Coot 0.89, Phenix 1.18.2, MotionCor2.1, CTFFIND4.1, UCSF Chimera 1.15, UCSF ChimeraX 1.4, crYOLO 1.7.4, MolProbity 4.1, Prism 9,Amber20 |

For manuscripts utilizing custom algorithms or software that are central to the research but not yet described in published literature, software must be made available to editors and reviewers. We strongly encourage code deposition in a community repository (e.g. GitHub). See the Nature Portfolio guidelines for submitting code & software for further information.

## Data

Policy information about availability of data

All manuscripts must include a data availability statement. This statement should provide the following information, where applicable:

- Accession codes, unique identifiers, or web links for publicly available datasets
- A description of any restrictions on data availability
- For clinical datasets or third party data, please ensure that the statement adheres to our policy

All data produced or analyzed in this study are included in the main text or the supplementary figures/tables. Source data are provided with this paper. The cryo-EM density maps and atomic coordinates have been deposited in the Electron Microscopy Data Bank (EMDB) and Protein Data Bank (PDB) under accession numbers EMD-36712 [https://www.ebi.ac.uk/emdb/EMD-36712] and 8JXT [http://doi.org/10.2210/pdb8JXT/pdb] for H4R/Histamine/Gi complex; EMD-36716 [https://

www.ebi.ac.uk/emdb/EMD-36716] and 8JXX [http://doi.org/10.2210/pdb8JXX/pdb] for H4R/Clobenpropit/Gi complex; EMD-36715 [https://www.ebi.ac.uk/emdb/EMD-36715] and 8JXW [http://doi.org/10.2210/pdb8JXW/pdb] for H4R/VUF6884/Gi complex and EMD-36714 [https://www.ebi.ac.uk/emdb/EMD-36714] and 8JXV [http://doi.org/10.2210/pdb8JXV/pdb] for H4R/Clozapine/Gi complex. The MD simulation data were deposited to Zenodo [https://doi.org/10.5281/zenodo.10802634].

## Research involving human participants, their data, or biological material

Policy information about studies with human participants or human data. See also policy information about sex, gender (identity/presentation), and sexual orientation and race, ethnicity and racism.

| | |
|---|---|
| Reporting on sex and gender | not applicable |
| Reporting on race, ethnicity, or other socially relevant groupings | not applicable |
| Population characteristics | not applicable |
| Recruitment | not applicable |
| Ethics oversight | not applicable |

Note that full information on the approval of the study protocol must also be provided in the manuscript.

# Field-specific reporting

Please select the one below that is the best fit for your research. If you are not sure, read the appropriate sections before making your selection.

☒ Life sciences ☐ Behavioural & social sciences ☐ Ecological, evolutionary & environmental sciences

For a reference copy of the document with all sections, see nature.com/documents/nr-reporting-summary-flat.pdf

# Life sciences study design

All studies must disclose on these points even when the disclosure is negative.

| | |
|---|---|
| Sample size | For all binding and functional assays, based on previous similar study (PMID: 36127364,36309016,35241677), three to five independent experiments (n=3-5) are carried out and sufficient. See figure legends for detail. |
| Data exclusions | No data were excluded |
| Replication | all experiments were repeated at least three times independently, all attempts at replication were successful. |
| Randomization | No Randomization was attempted or needed. Randomization was not necessary as the independent variables to be tested were sufficient for functional interpretation within this study. This is not a clinical trial or animal study that is dependent on randomization. |
| Blinding | Blinding was not necessary for structural determination as in this case, cryo-EM captured conformations representing a large amount of individual particles, or most time the major classes of all particles. Also, blinding is not necessary for functional analysis of this study as in this case, each mutations has it own distinct space coordinates. All experimental data acquired in this study are subjected to statistical analysis when necessary. |

# Reporting for specific materials, systems and methods

We require information from authors about some types of materials, experimental systems and methods used in many studies. Here, indicate whether each material, system or method listed is relevant to your study. If you are not sure if a list item applies to your research, read the appropriate section before selecting a response.

## Materials & experimental systems

| n/a | Involved in the study |
| --- | --- |
| ☐ | ☒ Antibodies |
| ☐ | ☒ Eukaryotic cell lines |
| ☒ | ☐ Palaeontology and archaeology |
| ☒ | ☐ Animals and other organisms |
| ☒ | ☐ Clinical data |
| ☒ | ☐ Dual use research of concern |
| ☒ | ☐ Plants |

## Methods

| n/a | Involved in the study |
| --- | --- |
| ☒ | ☐ ChIP-seq |
| ☒ | ☐ Flow cytometry |
| ☒ | ☐ MRI-based neuroimaging |

# Antibodies

Antibodies used

Anti-HA, Sigma,Cat# 11867423001; anti-rat-HRP, Sigma, Cat# AP136P

Validation

The validation of the The antibodies were validated by the manufactures in their specific data sheets. Here is the specificity from the manufactures data sheet: Anti-HA High Affinity (3F10) specifically recognizes the HA peptide sequence [YPYDVPDYA] derived from the influenza hemagglutinin protein. The antibody recognizes its antigenic determinant even when the HA peptide epitope is introduced into unrelated recombinant proteins by a technique known as epitope tagging.

# Eukaryotic cell lines

Policy information about cell lines and Sex and Gender in Research

Cell line source(s)

sf9 cell line, Invitorgen cat#11496-015; HEK293T cell line, ATCC, Cat# CRL-1573

Authentication

Cells have not been authenticated after purchase

Mycoplasma contamination

All cell lines tested are negative for mycoplasma contamination

Commonly misidentified lines
(See ICLAC register)

No commonly misidentified cell lines were used
